# On the Different Abilities of Cross-Sample Entropy and K-Nearest-Neighbor Cross-Unpredictability in Assessing Dynamic Cardiorespiratory and Cerebrovascular Interactions

**DOI:** 10.3390/e25040599

**Published:** 2023-04-01

**Authors:** Alberto Porta, Vlasta Bari, Francesca Gelpi, Beatrice Cairo, Beatrice De Maria, Davide Tonon, Gianluca Rossato, Luca Faes

**Affiliations:** 1 Department of Biomedical Sciences for Health, University of Milan, 20133 Milan, Italy; vlasta.bari@unimi.it (V.B.); francesca.gelpi@unimi.it (F.G.); beatrice.cairo@unimi.it (B.C.); 2 Department of Cardiothoracic, Vascular Anesthesia and Intensive Care, IRCCS Policlinico San Donato, 20097 Milan, Italy; 3 IRCCS Istituti Clinici Scientifici Maugeri, 20138 Milan, Italy; beatrice.demaria@icsmaugeri.it; 4 Department of Neurology, IRCCS Sacro Cuore Don Calabria Hospital, 37024 Verona, Italy; davide.tonon@sacrocuore.it (D.T.); gianluca.rossato@sacrocuore.it (G.R.); 5 Department of Engineering, University of Palermo, 90128 Palermo, Italy; luca.faes@unipa.it

**Keywords:** model-free time series analysis, causality, coupling strength, cardiac control, cerebral autoregulation, heart rate variability, blood flow, arterial pressure, autonomic nervous system, controlled breathing, head-up tilt

## Abstract

Nonlinear markers of coupling strength are often utilized to typify cardiorespiratory and cerebrovascular regulations. The computation of these indices requires techniques describing nonlinear interactions between respiration (R) and heart period (HP) and between mean arterial pressure (MAP) and mean cerebral blood velocity (MCBv). We compared two model-free methods for the assessment of dynamic HP–R and MCBv–MAP interactions, namely the cross-sample entropy (CSampEn) and k-nearest-neighbor cross-unpredictability (KNNCUP). Comparison was carried out first over simulations generated by linear and nonlinear unidirectional causal, bidirectional linear causal, and lag-zero linear noncausal models, and then over experimental data acquired from 19 subjects at supine rest during spontaneous breathing and controlled respiration at 10, 15, and 20 ${breaths\cdot minute}^{-1}$ as well as from 13 subjects at supine rest and during 60° head-up tilt. Linear markers were computed for comparison. We found that: (i) over simulations, CSampEn and KNNCUP exhibit different abilities in evaluating coupling strength; (ii) KNNCUP is more reliable than CSampEn when interactions occur according to a causal structure, while performances are similar in noncausal models; (iii) in healthy subjects, KNNCUP is more powerful in characterizing cardiorespiratory and cerebrovascular variability interactions than CSampEn and linear markers. We recommend KNNCUP for quantifying cardiorespiratory and cerebrovascular coupling.

## 1. Introduction

There are an increasing number of studies assessing the degree of coupling between respiration (R) and heart period (HP) [1,2,3,4,5,6,7] and between mean arterial pressure (MAP) and mean cerebral blood velocity (MCBv) [8,9,10,11,12,13,14]. This interest is justified by clinical relevance: indeed, the degree of HP–R coupling is taken as a marker of vagal control being inherently normalized by breathing activity [1,2,3,4,5,6,7], while the strength of the MCBv–MAP relationship is an indicator of the efficiency of dynamic cerebral autoregulation (dCA) [8,9,10,11,12,13,14].

The dynamic interactions between R and HP and between MAP and MCBv feature common characteristics: (i) they are inherently nonlinear; (ii) their strength is between the maximum and the minimum values found in the presence of full coupling and perfect uncoupling, respectively.

Nonlinearities between R and HP result from the periodical inhibition of aortic arch, carotid sinus, pulmonary, and atrial stretch receptor activity with R [15,16,17], reciprocal influences between sympathetic and vagal activities [18,19,20], variations of HP variability with both breathing rate and depth [21,22,23], phase locking between heartbeat and R rhythm [24,25,26], and the gating activity of respiratory centers over vagal and sympathetic outflows [27,28,29,30]. On the other hand, nonlinearities between MAP and MCBv are the result of the shape of the static characteristic of cerebrovascular autoregulation [31], different MCBv responses to positive and negative MAP variations [32,33], respiratory modulations of the MCBv–MAP relationship [34], effects of intracranial pressure on the critical closing pressure [35], and nonlinear influences of the autonomic control, especially over long time scales [36].

The imperfect association between R and HP is the result of the huge number of control mechanisms adapting HP regardless of R [37,38] and the variable cardiorespiratory phase locking ratio [24,25,39]. The imperfect association between MAP and MCBv is the consequence of the dCA aiming at keeping MCBv constant by buffering MAP changes with suitable adaptations to vessel diameter [8,9,10,11].

Given the abovementioned features of the HP–R and MCBv–MAP couplings, nonlinear tools should be preferred for the assessment of the strength of the interactions and these methods should be reliable in the presence of weak relationships. The reliability of these tools is a critical issue because modifications to HP–R and MCBv–MAP coupling strength are hallmarks of pathology [1,2,8,9].

Cross-sample entropy (CSampEn) [40] and k-nearest-neighbor cross-unpredictability (KNNCUP) [41] are two methods devised to assess nonlinear interactions between two time series. The ability of CSampEn and KNNCUP to describe nonlinear dynamics lies in their model-free nature that does not impose any form to the underlying relationship between the observed series. After reconstructing the dynamics of the two series in two distinct state spaces built via the time-delay embedding procedure, CSampEn estimates the negative logarithm of the conditional probability that, if two patterns of length *m* − 1 are similar, they remain alike after including one additional future value: the higher the conditional probability, the smaller the negative logarithm, the stronger the relationship and the coupling between the two series [40]. KNNCUP exploits the *k* patterns built over *m* − 1 past samples of the driver signal, namely R and MAP in our application, selected among others for their similarity with the reference vector, to predict the current value of the target signal, namely HP and MCBv, respectively: the lower the unpredictability of the target using the driver, the stronger the coupling from the driver to the target [41].

The aim of the present study is to compare the abilities of CSampEn and KNNCUP to characterize cardiorespiratory and cerebrovascular couplings via the analysis of the variability of R and HP and of MAP and MCBv. The two tools were first tested over simulations generated by linear and nonlinear unidirectional causal, linear bidirectional causal, and lag-zero linear noncausal models to better understand eventual differences in assessing dynamic HP–R and MCBv–MAP interactions. CSampEn and KNNCUP were then assessed in healthy subjects under experimental protocols inducing modifications of the cardiorespiratory and cerebrovascular controls, namely controlled breathing (CB) at different breathing rates [42,43] and head-up tilt (HUT) with a tilt table inclination of 60° [44,45]. Results of CSampEn and KNNCUP were compared with linear markers of coupling [4,9]. Preliminary data were presented at the 44th Annual International Conference of the Engineering in Medicine and Biology Society [46].

## 2. Methods

### 2.1. Generalities for the Computation of CSampEn and KNNCUP

Given two systems X and Y, their joint activity is described by two realizations, *x* = {$x_{n}$, 1 ≤ *n* ≤ *N*} and *y* = {$y_{n}$, 1 ≤ *n* ≤ *N*}, of the stochastic processes *X* and *Y*, respectively. The series x and y are usually collections of values recorded in experimental sessions designed to probe into the interactions between X and Y. We denote: (i) the current value of y as $y_{i}$; (ii) the pattern formed by the *m* − 1 past values of $y_{i}$ as $y_{i}^{-}=\left[\begin{array}{ccc} y_{i-1} & \ldots & y_{i-m+1} \end{array}\right]$; and (iii) the *m*-dimensional vector obtained by concatenating $y_{i}$ with $y_{i}^{-}$ as $y_{i}=\left[\begin{array}{cc} \begin{array}{cc} y_{i} & y_{i-1} \end{array} & \begin{array}{cc} \ldots & y_{i-m+1} \end{array} \end{array}\right]$. We remark that $y_{i}^{-}$ and $y_{i}$ can be interpreted indifferently as patterns of length, respectively, and *m* − 1 and *m* in the time domain or points of, respectively, the (*m* − 1)- and *m*-dimensional state phase spaces built uniformly using the method of lagged coordinates in the state phase domain. Analogously, we define $x_{j}$, $x_{j}^{-}=\left[\begin{array}{ccc} x_{j-1} & \ldots & x_{j-m+1} \end{array}\right]$, and $x_{j}=\left[\begin{array}{cc} \begin{array}{cc} x_{j} & x_{j-1} \end{array} & \begin{array}{cc} \ldots & x_{j-m+1} \end{array} \end{array}\right]$ the equivalent quantities computed over *x*. In order to characterize the relationship, if present, between X and Y, we indicate the probability that $y_{i}$ and $x_{j}$ are close in the *m*-dimensional state phase space within a tolerance *r* with $p(\left\|y_{i}-x_{j}\right\|\leq r)$ and with $p(\left\|y_{i}^{-}-x_{j}^{-}\right\|\leq r)$ the probability that $y_{i}^{-}$ and $x_{j}^{-}$ are closer than *r* in the (*m* − 1)-dimensional state phase space, where $\left\|\cdot \right\|$ is a metric to compute distance. In this study the adopted metric is the Euclidean norm.

### 2.2. CSampEn

CSampEn [40] is defined as the negative logarithm of the ratio of the averaged $p\left(\left\|y_{i}-x_{j}\right\|\leq r\right)$ to the averaged $p(\left\|y_{i}^{-}-x_{j}^{-}\right\|\leq r)$ as

(1) $$\mathrm{CSampEn}(m,\;r,\;N)=-log\left(\frac{\langle p\left(\left\|y_{i}-x_{j}\right\|\leq r\right)\rangle }{\langle p\left(\left\|y_{i}^{-}-x_{j}^{-}\right\|\leq r\right)\rangle }\right),$$

where $\langle \cdot \rangle$ performs the time average over all the reference vectors built from *x* and $log\left(\cdot \right)$ is the natural logarithm. $p\left(\left\|y_{i}-x_{j}\right\|\leq r\right)$ and $p(\left\|y_{i}^{-}-x_{j}^{-}\right\|\leq r)$ are estimated by counting the number of $y_{i}$ closer than *r* to $x_{j}$ for *i* = *m*, …, *N* and the number of $y_{i}^{-}$ closer than *r* to $x_{j}^{-}$ for *i* = *m* − 1, …, *N* − 1 and by dividing them by *N* – *m* + 1. The unfortunate case was that $\langle p\left(\left\|y_{i}-x_{j}\right\|\leq r\right)\rangle$ = 0 and/or $\langle p(\left\|y_{i}^{-}-x_{j}^{-}\right\|\leq r)\rangle =0$ were never observed in our study given the adopted values of *m* and *N*. CSampEn is a measure of the negative logarithm of the conditional probability that two points remain close in the *m*-dimensional phase space given that they are close in the (*m* − 1)-dimensional phase space: the closer to 1 the conditional probability, the lower the uncertainty associated with the reciprocal position of patterns in the *m*-dimensional phase space, the smaller the CSampEn, the stronger the relationship between X and Y.

### 2.3. KNNCUP

Cross-unpredictability (CUP) searches for the dependency $f\left(\cdot \right)$ of the future value $y_{i+\tau }$ of the dynamics of the target Y on *m* − 1 past samples $x_{i}^{-}$ of the dynamics of the driver X [41,47], where *τ* is the prediction horizon [14]. $y_{i+\tau }$ is usually labelled as the image of $x_{i}^{-}$ through $f\left(\cdot \right)$ and $x_{i}^{-}$ is the reference vector for the search of its *k* nearest neighbors. Local CUP approach approximates $f\left(\cdot \right)$ in a region around $x_{i}^{-}$ set by its *k* nearest neighbors [48]. Given the adopted metric for evaluating distances in the phase space, the region is a hypersphere and $x_{i}^{-}$ is its center. The *k* nearest neighbors of the reference vector $x_{i}^{-}$ were utilized to predict $y_{i+\tau }$. The prediction of $y_{i+\tau }$, namely ${\hat{y}}_{i+\tau }$, is defined as the weighted mean of the images of the *k* nearest neighbors of $x_{i}^{-}$, where the weights are the inverse of their distance from the reference vector. Vectors at zero distance from $x_{i}^{-}$ were excluded from the set of *k* nearest neighbors. ${\hat{y}}_{i+\tau }$ was computed as a function of time *i*, thus providing the predicted series $\hat{y}$. The degree of unpredictability of Y given X is measured via the CUP function defined as 1 − $\rho^{2}$, where $\rho^{2}$ is the squared correlation coefficient between y and $\hat{y}$. CUP is bounded between 0 and 1, where 0 indicates that *y* can be perfectly predicted from *x* and 1 indicates complete unpredictability of *y* based on *x*. CUP depends on *m*. The course of CUP with *m* was the result of two opposite tendencies [49]: (i) prediction of y given x might improve and CUP might decrease because longer patterns built over x bring more information about the future behavior of y; (ii) at high *m*, the *k* nearest neighbors tend to be far away from the reference vector due to the spreading of the vectors in the phase space and this effect raises CUP. The minimum of the CUP function over *m* was taken as a measure of the uncoupling between x and y [41] and referred to as the CUP index (CUPI): the closer to 0 the CUPI, the greater the ability to predict y based on x, the stronger the relationship between X and Y.

## 3. Simulations

### 3.1. Graded Unidirectional and Bidirectional Causal Couplings

We simulated bivariate autoregressive (BAR) processes whose components feature different degrees of coupling and causal relationships. The BAR process is defined as

(2) $$X\left(n\right)={2\rho }_{1}\cdot \left[c_{1}\cdot Y\left(n-1\right)+\left({1-c}_{1}\right)\cdot X\left(n-1\right)\right]\cdot cos\varphi_{1}-\rho_{1}^{2}\cdot X\left(n-2\right)+W_{1}\left(n\right)\;Y\left(n\right)={2\rho }_{2}\cdot \left[c_{2}\cdot X\left(n-1\right)+\left({1-c}_{2}\right)\cdot Y\left(n-1\right)\right]\cdot cos\varphi_{2}-\rho_{2}^{2}\cdot Y\left(n-2\right)+W_{2}\left(n\right)$$

where $W_{1}$ and $W_{2}$ are Gaussian white noises with zero mean and variances assigned such that X and Y exhibit unit variance. If $c_{1}=0$ and $c_{2}=0$, X and Y are two uncoupled second-order autoregressive [AR(2)] processes. If $c_{1}=0$ and $c_{2}\neq 0$, the directionality of the interactions is from X to Y (i.e., unidirectional causal model). If $c_{1}\neq 0$ and $c_{2}\neq 0$, the directionality of the interactions is from X to Y and vice versa (i.e., bidirectional causal model). In the uncoupled condition, the two processes X to Y were set to feature dominant rhythms according to two configurations: (i) $\rho_{1}=\rho_{2}=0.8$ with phases $\varphi_{1}=\pm 3\pi /5$ and $\varphi_{2}=\pm 3\pi /5$ corresponding to an oscillation at normalized frequency $f_{1}=0.3\;{cycles\cdot sample}^{-1}$ and $f_{2}=0.3$ ${cycles\cdot sample}^{-1}$, thus simulating a dominant rhythm in the high frequency (HF) band typical of spontaneous respiration driving an HP rhythm at the same frequency with a mean HP equal to 1 s; (ii) $\rho_{1}=\rho_{2}=0.8$ with phases $\varphi_{1}=\pm \pi /5$ and $\varphi_{2}=\pm \pi /5$ corresponding to an oscillation at normalized frequency $f_{1}=0.1\;{cycles\cdot sample}^{-1}$ and $f_{2}=0.1$ ${cycles\cdot sample}^{-1}$, thus leading to a dominant rhythm in the low frequency (LF) band typical of slow breathing driving an HP rhythm at the same frequency with a mean HP equal to 1 s. Coupling strengths between X and Y were varied according to the following setup: (i) $c_{1}=0$ and $c_{2}$ was varied incrementally from 0 to 1.0 in 0.1 steps, thus simulating unidirectional coupling from X to Y with incremental coupling strength (i.e., from the respiratory system to the heart or from the systemic to cerebral vasculature); $c_{1}=c_{2}=c$, and c was varied gradually from 0 to 1.0 in 0.1 steps, thus simulating bidirectional coupling from X to Y and vice versa with incremental coupling strength (i.e., from the respiratory system to the heart, or from the systemic to cerebral vasculature, and vice versa).

### 3.2. Graded Lag-Zero Noncausal Coupling

We simulated a bivariate process whose components featured different degrees of coupling in absence of any causal relationship. This bivariate process is obtained by corrupting an AR(2) process X with a Gaussian white noise $W_{2}$ with zero mean and standard deviation taken as a fraction of the standard deviation of X. The bivariate process is defined as

(3) $$Y\left(n\right)=X\left(n\right)+W_{2}\left(n\right),$$

where X is an AR(2) process with zero mean and unit standard deviation and $W_{2}$ is a Gaussian white noise with zero mean and standard deviation $1-c_{2}$. As in Section 3.1, X exhibits dominant rhythms according to two pole configurations: (i) $\rho_{1}=\rho_{2}=0.8$ with phases $\varphi_{1}=\pm 3\pi /5$ and $\varphi_{2}=\pm 3\pi /5$; (ii) $\rho_{1}=\rho_{2}=0.8$ with phases $\varphi_{1}=\pm \pi /5$ and $\varphi_{2}=\pm \pi /5$. Since interactions between X and Y occur at lag-zero, directionality is not set (i.e., the coupling is instantaneous), thus simulating noncausal interactions between X and Y. If $c_{2}=1$, X and Y are coincident and, assigned the outcome of the random experiment, the points $\left[x\left(n\right),y\left(n\right)\right]$ lie exactly on the diagonal line for *n* = 1, …, *N*, and the two processes X and Y are fully coupled. $c_{2}$ was varied incrementally from 0 to 1.0 in 0.1 steps, thus progressively decreasing the standard deviation of $W_{2}$ and increasing the coupling strength between X and Y.

### 3.3. Unidirectionally-Coupled Identical Logistic Maps

Nonlinear dynamics were simulated via logistic maps. We considered two unidirectionally-coupled identical logistic maps [50], described as

(4) $$X\left(n\right)=f[X\left(n-1\right)]\;Y\left(n\right)=c_{2}\cdot f[X\left(n-1\right)]+{(1-c}_{2})\cdot f[Y\left(n-1\right)],$$

with

(5) $$f[X\left(n-1\right)]=r\cdot X\left(n-1\right)\cdot [1-X\left(n-1\right)]\;f[Y\left(n-1\right)]=r\cdot Y\left(n-1\right)\cdot [1-Y\left(n-1\right)].$$

Thus, X and Y are deterministic signals obtained by iterating (4) starting from the initial conditions randomly chosen within the interval from 0.0 to 1.0. When $c_{2}=0,$ X and Y evolve independently of each other because their initial conditions are different according to the equations of the two logistic maps. Given that r=3.7, the two logistic maps are in chaotic regime. The coupling is unidirectional because Y does not affect X. The strength of the interactions from X to Y increases with $c_{2}$. The parameter $c_{2}$ was varied from 0 to 1.0 in 0.1 steps.

## 4. Experimental Protocol and Data Analysis

### 4.1. Ethical Statement

The R and HP series were extracted from a historical database built to evaluate changes in the cardiorespiratory coupling with the breathing rate in healthy subjects [42,43]. The MAP and MCBv series belonged to another historical database built to study the dCA during postural stimulus in healthy subjects [44,45]. All the original protocols were in keeping with the Declaration of Helsinki. The protocols were approved by the local ethical review board of the L. Sacco Hospital, Milan, Italy, and Sacro Cuore Don Calabria Hospital, Negrar, Italy, respectively, and authorized by the same structures. Written signed informed consent was obtained from all subjects. Physical examination and full neurological evaluation certified the healthy status of all the subjects. The subjects were not under pharmacological treatments interfering with cardiovascular and cerebrovascular controls. Subjects avoided caffeinated and alcoholic beverages and heavy physical exercises for 24 h before the study.

### 4.2. CB Protocol

Data were acquired from 19 healthy subjects (age: 27–35 years, median = 31 years; 8 males, 11 females) at rest in a supine position during spontaneous breathing (SB) and during CB at 10, 15, and 20 ${breaths\cdot minute}^{-1}$, labelled as CB10, CB15, and CB20, respectively. The period of SB always preceded the session of CB. The respiratory frequency of the CB was selected randomly. The subjects performed all the CB sessions. The timing of inspiratory and expiratory onsets was provided via a metronome and reinforced verbally by the experimenter. All the experimental sessions lasted 10 min. The subjects were not allowed to talk during the entire protocol. The electrocardiogram (ECG) was acquired from lead II via a bioamplifier (Marazza, Monza, Italy) and respiratory flow via a nasal thermistor (Marazza, Monza, Italy). Both signals were digitalized synchronously at 300 Hz by an analog-to-digital 12-bit board (National Instruments, Austin, TX, USA) plugged into a personal computer. From the ECG, we derived the beat-to-beat variability series of the HP and a downsampled version of the R signal. After identifying the R-wave from the ECG, the inter-heartbeat interval between the *n*th and (*n* + 1)th R-wave peaks was taken as the *n*th HP. The R signal was sampled at the *n*th R-wave peak.

### 4.3. HUT Protocol

Data were acquired from 13 healthy subjects with no history of postural syncope (age: 27 ± 8 years; 5 males, 8 females). Subjects were instrumented to continuously monitor the ECG from lead II and noninvasive continuous arterial pressure (AP) from the middle finger of the nondominant arm (Finapres Medical Systems, Enschede, The Netherlands). The cerebral blood velocity (CBv), measured from the right, or left, middle cerebral artery through a transcranial Doppler device (Multi-Dop T, DWL, 2 MHz, Compumedics, San Juan Capistrano, CA, USA), was taken as a surrogate of cerebral blood flow [51]. The CBv signal was low-pass filtered with a sixth-order Butterworth filter with a cut-off frequency of 10 Hz. The signals were acquired synchronously at a sampling rate of 1000 Hz. The subjects underwent 10 min of recording at rest in a supine position (REST) followed by HUT with a tilt table inclination of 60°. Both sessions were under SB. Analyses were carried out 3 min after the HUT onset and within the first 10 min of HUT. None of the subjects exhibited presyncope signs. We utilized the detection of the R-wave on the ECG to trigger the process of identification of systolic AP (SAP) and diastolic AP (DAP). The *n*th SAP was taken as the maximum of the AP signal within the *n*th HP. The (*n* − 1)th DAP was defined as the minimum of the AP signal preceding the *n*th SAP. The *n*th MAP was computed as the ratio of the definite integral of AP between the occurrences of the (*n* − 1)th and *n*th DAP to the inter-diastolic interval. The same procedure was applied to CBv to calculate the MCBv. The fiducial points for the computation of MAP were utilized for the computation of the MCBv.

### 4.4. Time Domain Analysis

The HP, SAP, DAP, MAP, and MCBv series were manually checked and corrected in case of missing beats or misdetections. The effects of ectopic beats or isolated arrhythmic events were mitigated via linear interpolation. Synchronous sequences were randomly selected within the whole recordings. The length of the series was kept constant regardless of protocol and experimental condition. As to the CB protocol, we monitored the mean and variance of the HP series. These markers were labeled as *μ*_HP_ and *σ*^2^_HP_, respectively, and expressed in ms and ms^2^. In the CB protocol, *μ*_HP_ did not vary compared to SB, being 1010 ± 168, 989 ± 157, 1023 ± 162, and 1028 ± 162 during SB, CB10, CB15, and CB20, respectively, while *σ*^2^_HP_ increased solely during CB10 compared to SB, being 3368 ± 2622, 4784 ± 3356, 3705 ± 3091, and 2813 ± 2158 during SB, CB10, CB15, and CB20, respectively. As to the HUT series, we monitored the mean and variance of the MAP and MCBv. These indices were denoted as *μ*_MAP_, *σ*^2^_MAP_, *μ*_MCBv_, and *σ*^2^_MCBv_ and expressed in mmHg, mmHg^2^, cm·s^−1^ and cm^2^·s^−2^. In the HUT protocol, *μ*_MCBv_ decreased during HUT compared to REST, being 72 ± 23 and 62 ± 21, respectively, while *σ*^2^_MCBv_ increased from 19 ± 12 to 26 ± 16. In the HUT protocol, *μ*_MAP_ and *σ*^2^_MAP_ did not vary with HUT: *μ*_MAP_ was 99 ± 17 and 95 ± 12 at REST and during HUT, respectively, while *σ*^2^_MAP_ was 18 ± 21 and 19 ± 12.

### 4.5. Computation of a Linear Marker of Association between Time Series

Squared coherence function $K_{x,y}^{2}(f)$ provides an estimation of the degree of linear association between x and y as a function of the frequency f [4]. It is computed as the ratio of the square cross-spectrum modulus between x and y divided by the product of their power spectra. By definition, $K_{x,y}^{2}(f)$ is bounded between 0 and 1, where 0 and 1 indicate the minimum and maximum correlation between x and y. A linear marker of the strength of the cardiorespiratory coupling is commonly computed by sampling $K_{R,HP}^{2}(f)$ at its peak in the HF band (i.e., from 0.15 to 0.4 Hz) [4,7]. This marker is indicated as $K_{R,HP}^{2}(HF)$ in the following. A linear marker of the strength of the cerebrovascular coupling is routinely computed by sampling $K_{MAP,MCBv}^{2}(f)$ at its peak in the very LF (VLF, from 0.02 to 0.07 Hz), LF (from 0.07 to 0.15 Hz), and HF (i.e., from 0.15 to 0.4 Hz) bands [8,9]. These markers are indicated as $K_{MAP,MCBv}^{2}(VLF)$, $K_{MAP,MCBv}^{2}\left(LF\right),$ and $K_{MAP,MCBv}^{2}(HF)$ in the following. The superior limit of the LF band and the inferior limit of the HF were modified compared to the original definition [8] to account for possible slow respiratory rhythms [12]. $K_{R,HP}^{2}(f)$ and $K_{MAP,MCBv}^{2}(f)$ were estimated according to a bivariate AR model [7]. The model order was fixed to 10, and the coefficients of the bivariate AR model were identified via the least squares approach [7].

### 4.6. Computation of CSampEn and KNNCUP

Since the aim of the study was to explore the physiological mechanisms responsible for short-term control of HP and MCBv, the length *N* of the series was set to 256 [8,52]. The series were first linearly detrended and then normalized to have zero mean and unit variance. Cardiorespiratory coupling was assessed with x=R and y=HP, while the cerebrovascular link was evaluated using x=MAP and y=MCBv. CSampEn was computed over the normalized series with *m* = 3 and *r* = 0.2 [14]. KNNCUP was performed with *k* = 30 [41]. Over simulated data, KNNCUP was computed with time horizon τ=−1 in the case of lag-zero noncausal model and with time horizon τ=0 in the case of unidirectional and bidirectional causal models. Over experimental data, KNNCUP was carried out with τ=−1 in agreement with the fast vagal actions responsible for the respiratory sinus arrhythmia [23,53] and fast resistive component of the MCBv–MAP relationship [54,55].

### 4.7. Statistical Analysis

One-way repeated measures analysis of variance (Dunnett’s test for multiple comparisons), or Friedman repeated measures analysis of variance on ranks if appropriate (Dunnett’s test for multiple comparisons), was utilized to check the effect of CB versus SB. A paired *t*-test, or a Wilcoxon signed rank test when appropriate, was applied to check the effect of HUT. Statistical analysis was carried out using a commercial statistical program (Sigmaplot, v.14.0, Systat Software, Inc., Chicago, IL, USA). A *p* < 0.05 was always considered statistically significant.

## 5. Results

### 5.1. Results on Simulations

**Figure 1:**
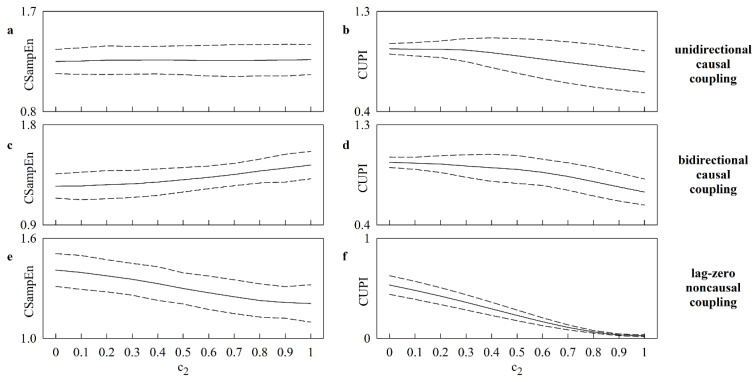
The line plots show the mean (solid line) and the confidence interval of two standard deviations about the mean (dashed lines) of CSampEn (**a**,**c**,**e**) and CUPI (**b**,**d**,**f**) as a function of $c_{2}$. The results of simulations generated via linear unidirectional causal (i.e., $c_{1}=0$), linear bidirectional causal (i.e., $c_{1}=c_{2}$), and lag-zero linear noncausal models are shown in (**a**,**b**), (**c**,**d**), and (**e**,**f**), respectively. The processes exhibit a dominant HF rhythm. The curves were built over 20 realizations of X and Y.

The line plot of Figure 1 shows the mean (solid line) and the confidence interval of two standard deviations about the mean (dashed lines) of CSampEn (Figure 1a,c,e) and CUPI (Figure 1b,d,f) computed over 20 realizations of X and Y. Simulations were generated via linear unidirectional causal (Figure 1a,b), linear bidirectional causal (Figure 1c,d) and lag-zero linear noncausal (Figure 1e,f) models. The simulated series featured a dominant HF rhythm. Regardless of the type of simulations, the expectation is that the coupling strength increases progressively with $c_{2}$. According to this expectation, CUPI decreased gradually with $c_{2}$, and this result held in simulations of linear unidirectional causal (Figure 1b), linear bidirectional causal (Figure 1d), and lag-zero linear noncausal (Figure 1f) couplings. Conversely, CSampEn declined gradually solely in simulations relevant to the lag-zero linear noncausal model (Figure 1e), being stable over unidirectional causal coupling (Figure 1a) and paradoxically increasing in bidirectional causal interactions (Figure 1c). Results stress the limited ability of CSampEn and the more reliable performance of CUPI.

**Figure 2:**
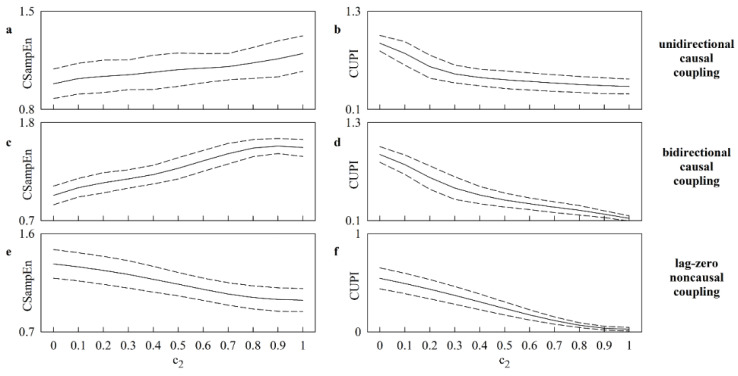
The line plots show the mean (solid line) and the confidence interval of two standard deviations about the mean (dashed lines) of CSampEn (**a**,**c**,**e**) and CUPI (**b**,**d**,**f**) as a function of $c_{2}$. The results of simulations generated via linear unidirectional causal (i.e., $c_{1}=0$), linear bidirectional causal (i.e., $c_{1}=c_{2}$), and lag-zero linear noncausal models are shown in (**a**,**b**), (**c**,**d**), and (**e**,**f**), respectively. The processes exhibit a dominant LF rhythm. The curves were built over 20 realizations of X and Y.

Like Figure 1, Figure 2 shows the results of simulations generated via linear unidirectional causal (Figure 2a,b), linear bidirectional causal (Figure 2c,d) and lag-zero linear noncausal (Figure 2e,f) models. However, the results are relevant to 20 realizations of X and Y featuring a dominant LF rhythm. The results stress the greater ability of CUPI in following the increased coupling strength with $c_{2}$. Indeed, CUPI gradually decreased with $c_{2}$ regarless of the type of simulations (Figure 2b,d,f). CSampEn exhibited bad performance over causal models (Figure 2a,c) and good performance in the case of lag-zero linear noncausal interactions (Figure 2e).

**Figure 3:**
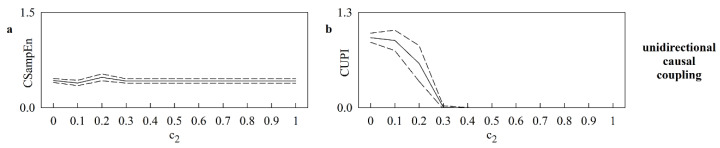
The line plots show the mean (solid line) and the confidence interval of two standard deviations about the mean (dashed lines) of CSampEn (**a**) and CUPI (**b**) as a function of $c_{2}$. The results are relevant to unidirectionally-coupled identical logistic maps. The curves were built over 20 pairs of X and Y generated according to different initial conditions.

Figure 3 shows the mean (solid line) and the confidence interval of two standard deviations about the mean (dashed lines) of CSampEn (Figure 3a) and CUPI (Figure 3b) computed over dynamics generated via unidirectionally-coupled identical logistic maps while varying $c_{2}$. The curves were built over 20 pairs of signals generated according to different initial conditions. CUPI decreased to 0 with $c_{2}$ and reached 0 when X and Y synchronized (Figure 3b). Conversely, CSampEn remained stable with $c_{2}$, and this result was the consequence of the inability of CSampEn to detect the situation of uncoupling when $c_{2}$ was close to 0 (Figure 3a).

### 5.2. Results on CB and HUT Protocols

**Figure 4:**
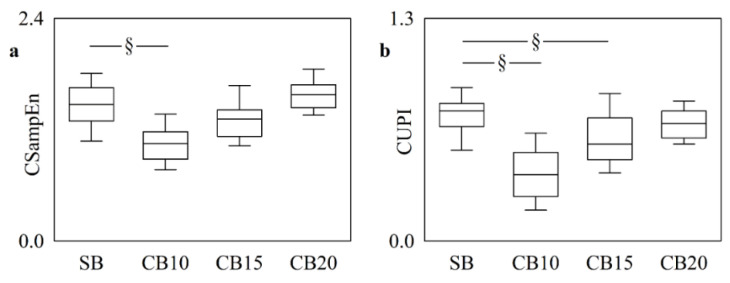
The vertical box-and-whisker plots show CSampEn (**a**) and CUPI (**b**) as a function of the experimental condition (i.e., SB, CB10, CB15, and CB20). The height of the box represents the distance between the first and third quartiles, with the median marked as a horizontal segment, and the whiskers denote the 5th and 95th percentiles. The symbol § indicates *p* < 0.05 versus SB.

Figure 4 shows CSampEn (Figure 4a) and CUPI (Figure 4b) computed in the CB protocol. CSampEn decreased significantly during CB10 compared to SB, thus suggesting that cardiorespiratory coupling increased at the slowest breathing rate. CUPI exhibited a similar trend, but its variation compared to SB was significant during both CB10 and CB15, thus making more evident the dependence of the cardiorespiratory coupling strength on the respiratory rate during CB.

**Figure 5:**
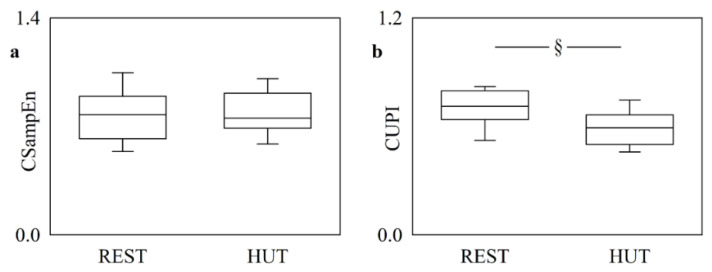
The vertical box-and-whisker plots show CSampEn (**a**) and CUPI (**b**) as a function of the experimental condition (i.e., REST and HUT). The height of the box represents the distance between the first and third quartiles, with the median marked as a horizontal segment, and the whiskers denote the 5th and 95th percentiles. The symbol § indicates *p* < 0.05 versus REST.

Figure 5 shows CSampEn (Figure 5a) and CUPI (Figure 5b) computed in the HUT protocol. The two markers exhibited striking differences with the experimental condition. Indeed, CUPI decreased significantly during HUT, thus suggesting an increase of the cerebrovascular coupling strength, while CSampEn remained stable, thus suggesting a certain stiffness in following modifications of the cerebrovascular coupling with the experimental condition.

**Figure 6:**
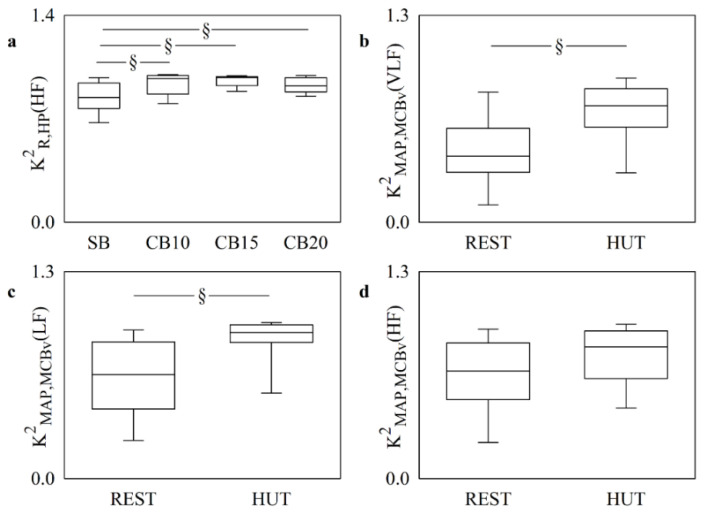
The vertical box-and-whisker plots show the K^2^ marker computed between R and HP in the HF band in the CB protocol (**a**) and the K^2^ markers computed between MAP and MCBv in the VLF (**b**), LF (**c**), and HF (**d**) bands in the HUT protocol. The height of the box represents the distance between the first and third quartiles, with the median marked as a horizontal segment, and the whiskers denote the 5th and 95th percentiles. The symbol § indicates *p* < 0.05 versus CB or REST.

Figure 6 shows the linear markers of cardiorespiratory (Figure 6a) and cerebrovascular (Figure 6b–d) coupling calculated in the CB and HUT protocols, respectively. CB augmented $K_{R,HP}^{2}(HF)$ regardless of the rate of paced breathing (Figure 6a). $K_{MAP,MCBv}^{2}(VLF)$ and $K_{MAP,MCBv}^{2}(LF)$ increased during HUT, and this result indicated an increased strength of the cerebrovascular coupling (Figure 6b,c). Conversely, $K_{MAP,MCBv}^{2}(HF)$ did not vary during HUT (Figure 6d).

## 6. Discussion

The main findings of this study can be summarized as follows: (i) CSampEn and KNNCUP can quantify the modifications of coupling strength but with different abilities; (ii) KNNCUP is more reliable than CSampEn when interactions occur according to a causal structure, while performances are similar over a lag-zero linear noncausal model; (iii) in healthy subjects KNNCUP is more powerful in identifying the changes in cardiorespiratory and cerebrovascular coupling strength in response to experimental challenges than CSampEn and linear markers.

### 6.1. Assessing the Coupling Strength between Dynamic Systems via CSampEn and KNNCUP

The degree of interaction between dynamic systems X and Y is assessed in the present study via two model-free methods, namely CSampEn and KNNCUP.

CSampEn reconstructs the dynamics of X and Y in two separate embedding spaces built using the technique of delayed components and assesses the strength of the relationship between the embedding spaces by computing the negative logarithm of the conditional probability that two patterns generated, respectively, by X and Y that are close in the (*m* − 1)-dimensional embedding space remain close in the *m*-dimensional one. In other words, according to the philosophy of state space correspondence methods, if two patterns occupy the same region in the (*m* − 1)-dimensional embedding spaces, in presence of a significant coupling between X and Y, they do not move apart when a new component is added.

Therefore, although the model-free nature of CSampEn allows for the theoretical description of nonlinear interactions, strong nonlinearities that imply links among different regions of the phase space, such as those responsible for interactions among rhythms at different frequencies, cannot be reliably described and the method seems to be more suitable for describing relations occurring according to a 1:1 coupling ratio.

However, simulations provided in this study prove that, even in the presence of a 1:1 coupling ratio typically occurring when two processes with the same dominant oscillation are considered, CSampEn cannot detect the progressive modification of the coupling strength when interactions between X and Y are generated by a linear causal model. This conclusion held even in the case of a model simulating the interactions between two nonlinear deterministic signals generated by logistic maps in a chaotic regime. This limitation is related to the inability of CSampEn to interpret causality given that it is a symmetric metric under the reversal of the role between X and Y. The most regrettable feature of CSampEn is that it can suggest even the opposite trend with the coupling strength as shown in Figure 1c and Figure 2a,c. Solely in trivial simulations where two stochastic processes interact in absence of a causal structure (i.e., immediate interactions), CSampEn can detect the expected modifications with the coupling strength. The limited ability of CSampEn confirms that the exploitation of a metric based on conditional probability does not necessarily assure a causal approach [56].

KNNCUP assesses the relationship between X and Y using a completely different logic, namely the cross-predictability of Y from patterns taken from the past history of X. KNNCUP recalls model-based cross-conditional entropy even though the metric utilized to assess irregularity of future behaviors of Y given the activity of X is different from the evaluation of the logarithm of the variance of the innovation of Y given X [57]. Therefore, the pattern is created over the activity of X and the ability of this pattern to set future behaviors of Y is tested by measuring the ability of predicting y based on the knowledge of x. In addition to having the possibility to describe nonlinear interactions among rhythms at different frequencies according to the number of past samples of x utilized to predict y, KNNCUP could account for the causal structure of the mechanisms generating the interactions between X and Y. In addition to accounting for the directionality of the interactions, KNNCUP optimizes the embedding dimension and coarse graining via the search for the minimum over *m* [41,49] and the coverage of the embedding space with cells of different size according to the k-nearest-neighbor strategy [58], respectively. The most remarkable feature of KNNCUP is its robustness in indicating the expected modifications of the coupling strength regardless of the model structure (i.e., causal or noncausal), type of causal interactions (i.e., unidirectional or bidirectional), and signal feature (i.e., linear stochastic or nonlinear deterministic).

In conclusion, KNNCUP should be preferred to CSampEn as a model-free technique for the assessment of coupling strength even in the presence of interactions occurring at the same frequency.

### 6.2. Superior Ability of CUPI Compared to CSampEn in Evaluating Cardiorespiratory Coupling Strength

CSampEn and CUPI exhibited similar results in the CB protocol. Indeed, both methods detected an increase in cardiorespiratory coupling strength during CB10. This result is not surprising given that the same conclusion was reached via cross-conditional entropy based on a uniform quantization strategy for the computation of probabilities [42]. A linear approach based on $K_{R,HP}^{2}(f)$ was able to reach the same conclusion, but the trend with the breathing rate was not evident. Therefore, given the dependence of the cardiorespiratory coupling strength on the breathing rate, we recommend the use of a respiratory rate-matched control group in applications to pathological subjects. At first sight, this conclusion might be the consequence of the increase of the transfer function magnitude from R to HP while slowing the respiratory rate [4,21,22] resulting from the shape of the sinus node transfer function [59,60]. Conversely, since a rise in the transfer function magnitude is not necessarily accompanied by an increase of the coupling strength, the observed finding is more likely to be the consequence of a firmer pacing of the neural efferent activity operated by the respiratory centers [27,28,29,30]. Given that the influence of R on HP is very rapid [4,23,53] and produces dominant HP oscillations at the same frequency [22,23], the difficulties of CSampEn in assessing coupling strength are less evident in the CB protocol. However, CSampEn and CUPI are not fully equivalent. As a matter of fact, CSampEn exhibited a slightly lower statistical power in detecting the dependence of the cardiorespiratory coupling strength on the respiratory rate than CUPI. This finding suggests that some difficulties of SampEn might be linked to the presence of slower components in the responses of HP to R [5,61] and to the significant action in the reverse causal direction (i.e., from HP to R) [3,26,53]. In addition, since the likelihood of the cross-frequency coupling, for example, between the main respiratory rhythm and oscillations generated by baroreflex control loop [62,63] increases while slowing the breathing rate, the resulting impairment of the state space correspondence might have, more importantly, reduced the effectiveness of CSampEn compared to that of CUPI.

### 6.3. Superior Ability of CUPI Compared to CSampEn in Evaluating Cerebrovascular Coupling Strength

CUPI declined during HUT, thus indicating that postural challenge augmented the cerebrovascular coupling strength. This result might be the consequence of the raise of AP variability [64,65] that is not buffered with suitable changes to the vessel diameter and drives changes of MCBv via the pressure-to-flow relationship [66,67]. This consideration suggests a tendency toward a worsening of the dCA given that an increased association between MAP and MCBv variations has been observed during fainting [68] and in subjects with impaired dCA [9,69,70,71]. A linear approach based on $K_{MAP,MCBv}^{2}(f)$ suggested an increased cerebrovascular coupling strength as well. However, it is worth noting that the observed increase is likely to occur in a physiological range that does not compromise the dCA given that the quality of the MCBv response to a sustained step increase of MAP was found to be preserved during HUT [72,73]. Regardless of the magnitude of the changes, the robust assessment of the MCBv–MAP coupling strength is a critical issue. Indeed, traditional markers of MCBv–MAP association based on $K_{MAP,MCBv}^{2}(f)$ failed to detect an increased cerebrovascular coupling strength in pathological subjects with impaired dCA [74].

Remarkably, CSampEn did not decrease during HUT, thus stressing its limited ability to quantify the modifications of the cerebrovascular coupling strength during orthostatic challenge. This result might be the consequence of the causal structure of the dynamic MCBv–MAP interactions occurring in a closed loop [45,54,55,75] according to the pressure-to-flow link [76] and the Cushing-like pathway [77] mediated by the activity of the mechano-receptors of the brainstem [78] and fully operative under physiological modifications of intracranial pressure [79].

## 7. Conclusions

This study proves the different abilities of CSampEn and KNNCUP metrics in assessing the coupling strength between two time series and clarifies the conditions under which their behaviors diverge. In addition to the trivial disruption of the state space correspondence resulting from nonlinear interactions occurring according to the *n*:*m* coupling ratio, with *m* and *n* different from 1, even interactions occurring according to a causal structure are sufficient to limit the performance of CSampEn. We recommend the avoidance of the use of CSampEn to assess cardiorespiratory and cerebrovascular coupling from spontaneous variability, because CSampEn might be inadequate to describe the complex causal nature of these relationships [4,5,6,42,44,45,55,62,75,80]. Conversely, KNNCUP assures a more reliable nonlinear model-free framework to quantify cardiorespiratory and cerebrovascular variability interactions because its performances are less dependent on the structure of the underlying mechanism generating the dynamics, thus allowing the detection of a trend toward an increase of the cardiorespiratory coupling strength while slowing the breathing rate and the raise of the cerebrovascular coupling strength during the sympathetic activation and vagal withdrawal induced by an orthostatic challenge.

## Data Availability

The data presented in this study are available on request from the corresponding author (i.e., A.P.) upon permission of the IRCCS Policlinico San Donato. The data are not publicly available because they contain information that could compromise the privacy of research participants.

## References

[1] Elstad M., O’Callaghan E.L., Smith A.J., Ben-Tal A., Ramchandra R. (2018). Cardiorespiratory interactions in humans and animals: Rhythms for life. Am. J. Physiol..

[2] Penzel T., Kantelhardt J.W., Bartsch R.P., Riedl M., Kramer J., Wessel N., Garcia C., Glos M., Fietze I., Schöbel C. (2016). Modulations of heart rate, ECG, and cardio-respiratory coupling observed in polysomnography. Front. Physiol..

[3] Yana K., Saul J.P., Berger R.D., Perrott M.H., Cohen R.J. (1993). A time domain approach for the fluctuation analysis of heart rate related to instantaneous lung volume. IEEE Trans. Biomed. Eng..

[4] Saul J.P., Berger R.D., Chen M.H., Cohen R.J. (1989). Transfer function analysis of autonomic regulation II. Respiratory sinus arrhythmia. Am. J. Physiol..

[5] Triedman J.K., Perrott M.H., Cohen R.J., Saul J.P. (1995). Respiratory sinus arrhythmia: Time domain characterization using autoregressive moving average analysis. Am. J. Physiol..

[6] Porta A., Bassani T., Bari V., Tobaldini E., Takahashi A.C.M., Catai A.M., Montano N. (2012). Model-based assessment of baroreflex and cardiopulmonary couplings during graded head-up tilt. Comput. Biol. Med..

[7] Porta A., Maestri R., Bari V., De Maria B., Cairo B., Vaini E., La Rovere M.T., Pinna G.D. (2018). Paced breathing increases the redundancy of cardiorespiratory control in healthy individuals and chronic heart failure patients. Entropy.

[8] Claassen J.A., Meel-van den Abeelen A.S., Simpson D.M., Panerai R.B. (2016). and the international Cerebral Autoregulation Research Network (CARNet). Transfer function analysis of dynamic cerebral autoregulation: A white paper from the International Cerebral Autoregulation Research Network. J. Cereb. Blood Flow Metab..

[9] Giller C.A. (1990). The frequency-dependent behavior of cerebral autoregulation. Neurosurgery.

[10] Zhang R., Zuckerman J.H., Iwasaki K., Wilson T.E., Crandall C.G., Levine B.D. (2002). Autonomic neural control of dynamic cerebral autoregulation in humans. Circulation.

[11] Tzeng Y.C., Ainslie P.N., Cooke W.H., Peebles K.C., Willie C.K., Macrae B.A., Smirl J.D., Horsman H.M., Rickards C.A. (2012). Assessment of cerebral autoregulation: The quandary of quantification. Am. J. Physiol..

[12] Vaini E., Bari V., Fantinato A., Pistuddi V., Cairo B., De Maria B., Ranucci M., Porta A. (2019). Causality analysis reveals the link between cerebrovascular control and acute kidney dysfunction after coronary artery bypass grafting. Physiol. Meas..

[13] Hori D., Nomura Y., Ono M., Joshi B., Mandal K., Cameron D., Kocherginsky M., Hogue C.W. (2017). Optimal blood pressure during cardiopulmonary bypass defined by cerebral autoregulation monitoring. J. Thorac. Cardiovasc. Surg..

[14] Porta A., Gelpi F., Bari V., Cairo B., De Maria B., Panzetti C.M., Cornara N., Bertoldo E.G., Fiolo V., Callus E. (2022). Monitoring the evolution of asynchrony between mean arterial pressure and mean cerebral blood flow via cross-entropy methods. Entropy.

[15] Eckberg D.L., Kifle Y.T., Roberts V.L. (1980). Phase relationship between normal human respiration and baroreflex responsiveness. J. Physiol..

[16] Taha B.H., Simon P.M., Dempsey J.A., Skatrud J.B., Iber C. (1995). Respiratory sinus arrhythmia in humans: An obligatory role for vagal feedback from the lungs. J. Appl. Physiol..

[17] Crystal G.J., Salem M.R. (2012). The Bainbridge and the “reverse” Bainbridge reflexes: History, physiology, and clinical relevance. Anesth. Analg..

[18] Levy M.N. (1971). Sympathetic-parasympathetic interactions in the heart. Circ. Res..

[19] Kawada T., Sugimachi M., Shishido T., Miyano H., Sato T., Yoshimura R., Miyashita H., Nakahara T., Alexander J., Sunagawa K. (1999). Simultaneous identification of static and dynamic vagosympathetic interactions in regulating heart rate. Am. J. Physiol..

[20] Taylor J.A., Myers C.W., Halliwill J.R., Seidel H., Eckberg D.L. (2001). Sympathetic restraint of respiratory sinus arrhythmia: Implications for vagal-cardiac tone assessment in humans. Am. J. Physiol..

[21] Angelone A., Coulter N.A. (1964). Respiratory sinus arrhythmia: A frequency dependent phenomenon. J. Appl. Physiol..

[22] Hirsch J.A., Bishop B. (1981). Respiratory sinus arrhythmia in humans: How breathing pattern modulates heart rate. Am. J. Physiol..

[23] Eckberg D.L. (1983). Human sinus arrhythmia as an index of vagal cardiac outflow. J. Appl. Physiol..

[24] Schäfer G., Rosenblum M.G., Kurths J., Abel H.H. (1998). Heartbeat synchronized with ventilation. Nature.

[25] Cairo B., Martins de Abreu R., Bari V., Gelpi F., De Maria B., Rehder-Santos P., Sakaguchi C.A., Donisete da Silva C., De Favari Signini E., Catai A.M. (2021). Optimizing phase variability threshold for automated synchrogram analysis of cardiorespiratory interactions in amateur cyclists. Philos. Trans. R. Soc. A.

[26] Tzeng Y.C., Larsen P.D., Galletly D.C. (2003). Cardioventilatory coupling in resting human subjects. Exp. Physiol..

[27] Spyer K.M. (1995). Central nervous mechanisms responsible for cardio-respiratory homeostasis. Adv. Exp. Med. Biol..

[28] Gilbey M.P., Jordan D., Richter D.W., Spyer K.M. (1984). Synaptic mechanisms involved in the inspiratory modulation of vagal cardio-inhibitory neurones in the cat. J. Physiol..

[29] Seals D.R., Suwarno N.O., Dempsey J.A. (1990). Influence of lung volume on sympathetic nerve discharge in normal subjects. Circ. Res..

[30] Eckberg D.L., Nerhed C., Wallin B.G. (1985). Respiratory modulation of muscle sympathetic and vagal cardiac outflow in man. J. Physiol..

[31] Lassen N.A. (1959). Cerebral blood flow and oxygen consumption in man. Physiol. Rev..

[32] Aaslid R., Blaha M., Sviri G., Douville C.M., Newell D.W. (2007). Asymmetric dynamic cerebral autoregulatory response to cyclic stimuli. Stroke.

[33] Schmidt B., Klingelhofer J., Perkes I., Czosnyka M. (2009). Cerebral autoregulatory response depends on the direction of change in perfusion pressure. J. Neurotrauma.

[34] Bari V., Marchi A., De Maria B., Rossato G., Nollo G., Faes L., Porta A. (2016). Nonlinear effects of respiration on the crosstalk between cardiovascular and cerebrovascular control systems. Philos. Trans. R. Soc. A.

[35] Panerai R.B. (2003). The critical closing pressure of the cerebral circulation. Med. Eng. Phys..

[36] Hamner J.W., Tan C.O., Lee K., Cohen M.A., Taylor J.A. (2010). Sympathetic control of the cerebral vasculature in humans. Stroke.

[37] Gebber G.L., Barman S.M. (1977). Brain stem vasomotor circuits involved in the genesis and entrainment of sympathetic nervous rhythms. Progr. Brain Res..

[38] Marchi A., Bari V., De Maria B., Esler M., Lambert E., Baumert M., Porta A. (2016). Simultaneous characterization of sympathetic and cardiac arms of the baroreflex through sequence techniques during incremental head-up tilt. Front. Physiol..

[39] Bartsch R.P., Kantelhardt J.W., Penzel T., Havlin S. (2007). Experimental evidence for phase synchronization transitions in the human cardiorespiratory system. Phys. Rev. Lett..

[40] Richman J.S., Moorman J.R. (2000). Physiological time-series analysis using approximate entropy and sample entropy. Am. J. Physiol..

[41] Porta A., Faes L., Bari V., Marchi A., Bassani T., Nollo G., Perseguini N.M., Milan J., Minatel V., Borghi-Silva A. (2014). Effect of age on complexity and causality of the cardiovascular control: Comparison between model-based and model-free approaches. PLoS ONE.

[42] Porta A., Guzzetti S., Montano N., Pagani M., Somers V., Malliani A., Baselli G., Cerutti S. (2000). Information domain analysis of cardiovascular variability signals: Evaluation of regularity, synchronisation and co-ordination. Med. Biol. Eng. Comput..

[43] Porta A., Baselli G., Guzzetti S., Pagani M., Malliani A., Cerutti S. (2000). Prediction of short cardiovascular variability signals based on conditional distribution. IEEE Trans. Biomed. Eng..

[44] Faes L., Porta A., Rossato G., Adami A., Tonon D., Corica A., Nollo G. (2013). Investigating the mechanisms of cardiovascular and cerebrovascular regulation in orthostatic syncope through an information decomposition strategy. Auton. Neurosci. Basic Clin..

[45] Bari V., De Maria B., Mazzucco C.E., Rossato G., Tonon D., Nollo G., Faes L., Porta A. (2017). Cerebrovascular and cardiovascular variability interactions investigated through conditional joint transfer entropy in subjects prone to postural syncope. Physiol. Meas..

[46] Porta A., Bari V., Gelpi F., Cairo B., De Maria B., Tonon D., Rossato G., Faes L. (2022). Comparing cross-sample entropy and k-nearest-neighbor cross-predictability approaches for the evaluation of cardiorespiratory and cerebrovascular dynamic interactions. Proceedings of the 44th Annual International Conference of the IEEE EMBS.

[47] Schiff S.J., So P., Chang T., Burke R., Sauer T. (1996). Detecting dynamical interdependence and generalized synchrony through mutual prediction in a neural ensemble. Phys. Rev. E.

[48] Abarbanel H.D.I., Carroll T.L., Pecora L.M., Sidorowich J.J., Tsimring L.S. (1994). Predicting physical variables in time-delay embedding. Phys. Rev. E.

[49] Porta A., Castiglioni P., Bari V., Bassani T., Marchi A., Cividjian A., Quintin L., Di Rienzo M. (2013). K-nearest-neighbor conditional entropy approach for the assessment of short-term complexity of cardiovascular control. Physiol. Meas..

[50] Lloyd A.L. (1995). The coupled logistic map: A simple model for the effects of spatial heterogeneity on population dynamics. J. Theor. Biol..

[51] Aaslid R., Markwalder T.M., Nornes H. (1982). Noninvasive transcranial Doppler ultrasound recording of flow velocity in basal cerebral arteries. J. Neurosurg..

[52] Task Force of the European Society of Cardiology and the North American Society of Pacing and Electrophysiology (1996). Heart rate variability: Standards of measurement, physiological interpretation and clinical use. Circulation.

[53] Porta A., Castiglioni P., Di Rienzo M., Bassani T., Bari V., Faes L., Nollo G., Cividjan A., Quintin L. (2013). Cardiovascular control and time domain Granger causality: Insights from selective autonomic blockade. Philos. Trans. R. Soc. A.

[54] Bari V., Fantinato A., Vaini E., Gelpi F., Cairo B., De Maria B., Pistuddi V., Ranucci M., Porta A. (2021). Impact of propofol general anesthesia on cardiovascular and cerebrovascular closed loop variability interactions. Biomed. Signal Process. Control.

[55] Porta A., Gelpi F., Bari V., Cairo B., De Maria B., Tonon D., Rossato G., Ranucci M., Faes L. (2022). Categorizing the role of respiration in cardiovascular and cerebrovascular variability interactions. IEEE Trans. Biomed. Eng..

[56] Porta A., Bari V., De Maria B., Cairo B., Vaini E., Perseguini N.M., Milan-Mattos J., Rehder-Santos P., Minatel V., Takahashi A.C.M. (2018). Comparison between probabilistic and Wiener-Granger causality in assessing modifications of the cardiac baroreflex control with age. Physiol. Meas..

[57] Faes L., Porta A., Nollo G. (2015). Information decomposition in bivariate systems: Theory and application to cardiorespiratory dynamics. Entropy.

[58] Porta A., Guzzetti S., Furlan R., Gnecchi-Ruscone T., Montano N., Malliani A. (2007). Complexity and nonlinearity in short-term heart period variability: Comparison of methods based on local nonlinear prediction. IEEE Trans. Biomed. Eng..

[59] Chess G.F., Calaresu F.R. (1971). Frequency response model of vagal control of heart rate in the cat. Am. J. Physiol..

[60] Berger R.D., Saul J.P., Cohen R.J. (1989). Transfer function analysis of autonomic regulation I. Canine atrial rate response. Am. J. Physiol..

[61] Chen X., Mukkamala R. (2008). Selective quantification of the cardiac sympathetic and parasympathetic nervous systems by multisignal analysis of cardiorespiratory variability. Am. J. Physiol..

[62] Baselli G., Cerutti S., Badilini F., Biancardi L., Porta A., Pagani M., Lombardi F., Rimoldi O., Furlan R., Malliani A. (1994). Model for the assessment of heart period and arterial pressure variability interactions and respiratory influences. Med. Biol. Eng. Comput..

[63] Patton D.J., Triedman J.K., Perrott M.H., Vidian A.A., Saul J.P. (1996). Baroreflex gain: Characterization using autoregressive moving average analysis. Am. J. Physiol..

[64] Cooke W.H., Hoag J.B., Crossman A.A., Kuusela T.A., Tahvanainen K.U., Eckberg D.L. (1999). Human responses to upright tilt: A window on central autonomic integration. J. Physiol..

[65] Marchi A., Bari V., De Maria B., Esler M., Lambert E., Baumert M., Porta A. (2016). Calibrated variability of muscle sympathetic nerve activity during graded head-up tilt in humans and its link with noradrenaline data and cardiovascular rhythms. Am. J. Physiol..

[66] Zhang R., Zuckerman J.H., Levine B.D. (1998). Deterioration of cerebral autoregulation during orthostatic stress: Insights from the frequency domain. J. Appl. Physiol..

[67] Katsogridakis E., Bush G., Fan L., Birch A.A., Simpson D.M., Allen R., Potter J.F., Panerai R.B. (2013). Detection of impaired cerebral autoregulation improves by increasing arterial blood pressure variability. J. Cereb. Blood Flow Metab..

[68] Ocon A.J., Kulesa J., Clarke D., Taneja I., Medow M.S., Stewart J.M. (2009). Increased phase synchronization and decreased cerebral autoregulation during fainting in the young. Am. J. Physiol..

[69] Ocon A.J., Medow M.S., Taneja I., Clarke D., Stewart J.M. (2009). Decreased upright cerebral blood flow and cerebral autoregulation in normocapnic postural tachycardia syndrome. Am. J. Physiol..

[70] Castro P., Serrador J., Sorond F., Azevedo E., Rocha I. (2022). Sympathovagal imbalance in early ischemic stroke is linked to impaired cerebral autoregulation and increased infarct volumes. Auton. Neurosci. Basic Clin..

[71] Czosnyka M., Smielewski P., Kirkpatrick P., Menon D.K., Pickard J.D. (1996). Monitoring of cerebral autoregulation in head-injured patients. Stroke.

[72] Carey B.J., Manktelow B.N., Panerai R.B., Potter J.F. (2001). Cerebral autoregulatory responses to head-up tilt in normal subjects and patients with recurrent vasovagal syncope. Circulation.

[73] Gelpi F., Bari V., Cairo B., De Maria B., Tonon D., Rossato G., Faes L., Porta A. (2022). Dynamic cerebrovascular autoregulation in patients prone to postural syncope: Comparison of techniques assessing the autoregulation index from spontaneous variability series. Auton. Neurosci. Basic Clin..

[74] Caldas J.R., Panerai R.B., Haunton V.J., Almeida J.P., Ferreira G.S.R., Camara L., Nogueira R.C., Bor-Seng-Shu E., Oliveira M.L., Groehs R.R.V. (2017). Cerebral blood flow autoregulation in ischemic heart failure. Am. J. Physiol..

[75] Saleem S., Teal P.D., Howe C.A., Tymko M.M., Ainslie P.N., Tzeng Y.-C. (2018). Is the Cushing mechanism a dynamic blood pressure-stabilizing system? Insights from Granger causality analysis of spontaneous blood pressure and cerebral blood flow. Am. J. Physiol..

[76] Tzeng Y.C., MacRae B.A., Ainslie P.N., Chan G.S.H. (2014). Fundamental relationships between blood pressure and cerebral blood flow in humans. J. Appl. Physiol..

[77] Cushing H. (1901). Concerning a definitive regulatory mechanism of the vaso-motor centre which controls blood pressure during cerebral compression. Bull. Johns Hopkins Hosp..

[78] McBryde F.D., Malpas S.C., Paton J.F.R. (2017). Intracranial mechanisms for preserving brain blood flow in health and disease. Acta Physiol..

[79] Nakamura K., Osborn J.W., Cowley A.W. (1987). Pressor response to small elevations of cerebroventricular pressure in conscious rats. Hypertension.

[80] Panerai R.B., Dawson S.L., Potter J.F. (1999). Linear and nonlinear analysis of human dynamic cerebral autoregulation. Am. J. Physiol..

